# *MYBL2* mRNA expression as a potential biomarker of therapeutic response to genotoxic treatments in myelodysplastic syndrome

**DOI:** 10.18632/oncotarget.26477

**Published:** 2018-12-25

**Authors:** Rachel Bayley, Daniel Blakemore, Paloma García

**Affiliations:** Paloma García: Institute of Cancer and Genomic Sciences, College of Medical and Dental Sciences, University of Birmingham, Birmingham, United Kingdom

**Keywords:** DNA repair, stem cells, aging, blood disorders, MDS

Myelodysplastic syndromes represent a heterogeneous group of age-associated haematopoietic diseases, which originate from changes in the function of haematopoietic stem cells (HSC). The conditions are referred to as clonal neoplasms and are characterised mainly by abnormal blood cell maturation plus a high propensity of progression to acute myeloid leukaemia (AML) [1]. At present, the only available cure is stem cell transplantation, but in many cases, this is unfeasible. As such, low-risk MDS patients often only receive blood transfusions and erythropoietin to treat their anaemia and high-risk patients are treated with hypomethylating treatments such as azacytidine and perhaps intensive chemotherapy. Current MDS diagnosis involves assessment of multiple clinical parameters including cell counts, flow cytometry, chromosome analysis and mutational analysis of patient’s blood and bone marrow. Although these analyses provide clinicians with a broad picture of disease severity and measure factors that may affect disease progression, information regarding the mechanisms of disease development at the HSC level is distinctly lacking.

One aspect of MDS that remains unclear is which genetic mutations arising in the HSC population contribute significantly to disease pathogenesis. Over the last decade, next generation sequencing studies have shed light on this by investigating the clonality of MDS and made three main observations [2, 3, 4, 5]. These included the fact that (1) MDS founder clones contain hundreds of mutations which persist in patients progressing to AML, (2) many mutations are randomly acquired and not related to disease pathogenesis, and (3) recurrently mutated genes such as *DNMT3A, TET2, ASXL1, TP53* and *SF3B*, might be important for initiating the disease.

Although these studies identify commonly mutated genes in MDS, insight into the functional consequences of these mutations is often lacking. Furthermore, and with the exception of *SF3B*, mutations in these genes are often acquired during normal healthy ageing, making their use for diagnostic purposes difficult. Therefore, there is an unmet clinical need for the identification of new markers with diagnostic and prognostic value. In particular, identification of molecular markers that affect disease severity would allow the stratification of patients to potentially inform choice of treatment. To this end, our recent work has identified the RNA expression of the *MYBL2* gene in HSC as a critical determinant of disease prognosis and DNA double-strand break (DSB) repair in MDS patients [6].

MYBL2 is a transcription factor known to be important for maintaining genome integrity, and is located within the minimal commonly deleted region of the long arm of chromosome 20 (del20q), a frequent chromosomal abnormality present in MDS. We and others have shown previously that reduced *MYBL2* expression in CD34^+^ cells from MDS patients correlates with a poor prognosis even in patients with a normal karyotype, suggesting that this could contribute to disease pathology [7, 8]. This was confirmed by the observation that mice expressing low levels of Mybl2 develop haematological disorders during ageing that closely resemble human MDS [7, 8]. Taken together, these studies indicate that compromising *MYBL2* function plays a key role in the development and progression of MDS.

We have further investigated the mechanisms by which MYBL2 function could contribute to MDS development and uncovered an important role for MYBL2 in DNA DSB repair in HSC [6]. HSC obtained from Mybl2 haploinsufficient animals exhibited significant defects in DSB repair, caused by an inability to maintain ATM-dependent KAP-1 phosphorylation, which resulted in telomere fragility [6]. KAP-1 phosphorylation is essential for the repair of DSBs within regions of heterochromatin, suggesting a role for of Mybl2 within the ATM-dependent DNA damage response (DDR) crucial for maintaining genome stability. Interestingly, it has been recently reported that HSC from aged mice also display diminished ATM-dependent DDR signalling and reduced apoptotic priming, which facilitates the survival of genomically unstable stem cells [9]. In keeping with MDS arising from a population of stem cells with a compromised DDR, it has been reported that CD34^+^ cells from MDS patients exhibit a significant reduction in DNA repair gene expression [10], in particular those known to function with the ATM-dependent DDR, emphasizing a potential role for these genes in MDS pathogenesis.

**Figure 1:**
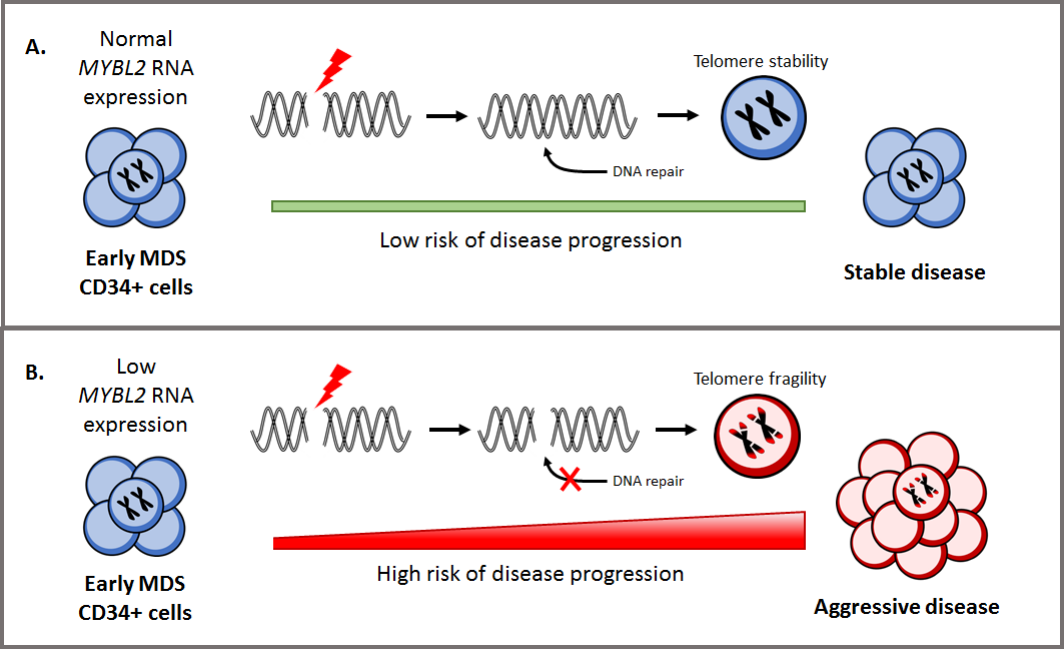
Influence of *MYBL2* expression on DNA repair and disease progression in myelodysplastic syndromes.

We also observed that MDS patients with low *MYBL2* levels exhibited a significant reduction in the expression of DNA repair genes present in the reactome DNA repair pathway (among them RAD51, BRCA1, BRCA2, XRCC5, MDC1) [6], indicating that DNA repair processes may be impaired. Moreover, MDS patient CD34^+^ cells with low *MYBL2* expression failed to efficiently repair DNA DSBs when compared to those with high *MYBL2* levels [6]. This correlation between *MYBL2* levels and DNA repair capacity indicates for the first time, that the poor prognosis associated with low *MYBL2* expression may arise from increased genome instability and the acquisition of mutations/chromosomal rearrangements that facilitate disease progression.

Given our findings, we suggest that HSC with a compromised MYBL2-dependent DDR can promote MDS development by facilitating telomere instability and inefficient repair of physiological DSBs. Taken together, these processes are known to contribute to oncogenic transformation in the HSC population, thus contributing to the pathology of MDS. Furthermore, the accumulation of incorrectly repaired DSBs in premalignant cells with sustained low levels of MYBL2 protein may also confer an increased susceptibility to disease progression. Overall, our work identifies the requirement of correct *MYBL2* levels to support DNA DSB repair in HSC, providing a molecular basis for the clinical phenotype. Our study has highlighted that *MYBL2* RNA expression could be used as a novel predictor of DNA repair capacity in CD34^+^ cells from MDS patients, and measuring this single gene enables the association of a wider and distinctive DNA repair gene signature. This could be a powerful clinical biomarker to allow better patient stratification, specifically to inform decisions regarding patient selection for transplantation or treatments which target DNA repair. MDS remains a complex and heterogeneous disease which will always require comprehensive genetic analysis. However, if in the future we can profile functional genetic changes similarly to low *MYBL2* expression, this will significantly improve our understanding, diagnosis and treatment of the disease in the ever-increasing ageing population.
